# Comparative analysis of landscape effects on spatial genetic structure of the big brown bat and one of its cimicid ectoparasites

**DOI:** 10.1002/ece3.3329

**Published:** 2017-09-06

**Authors:** Benoit Talbot, Maarten J. Vonhof, Hugh G. Broders, Brock Fenton, Nusha Keyghobadi

**Affiliations:** ^1^ Department of Biology University of Western Ontario London ON Canada; ^2^ Department of Biological Sciences Western Michigan University Kalamazoo MI USA; ^3^ Department of Biology University of Waterloo Waterloo ON Canada

**Keywords:** Chiroptera, Cimicidae, landscape ecology, landscape genetics, population genetics

## Abstract

Identification of landscape features that correlate with genetic structure permits understanding of factors that may influence gene flow in a species. Comparing effects of the landscape on a parasite and host provides potential insights into parasite‐host ecology. We compared fine‐scale spatial genetic structure between big brown bats (*Eptesicus fuscus*) and their cimicid ectoparasite (*Cimex adjunctus*; class Insecta) in the lower Great Lakes region of the United States, in an area of about 160,000 km^2^. We genotyped 142 big brown bat and 55 *C. adjunctus* samples at eight and seven microsatellite loci, respectively, and inferred effects of various types of land cover on the genetic structure of each species. We found significant associations between several land cover types and genetic distance in both species, although different land cover types were influential in each. Our results suggest that even in a parasite that is almost entirely reliant on its hosts for dispersal, land cover can affect gene flow differently than in the hosts, depending on key ecological aspects of both species.

## 1 INTRODUCTION

Landscape elements, and the composition and configuration of the surrounding landscape, affect dispersal and gene flow in a broad range of organisms (Manel & Holderegger, 2013; Manel, Schwartz, Luikart, & Taberlet, 2003; Storfer, Murphy, Spear, Holderegger, & Waits, 2010). Gene flow in turn affects genetic structure, such that less gene flow is associated with increased spatial structure and differentiation (Bohonak, 1999). The association between landscape variables and genetic structure or differentiation is now commonly used to infer which landscape elements may act to facilitate or impede gene flow (Storfer et al., 2007). Some studies have compared effects of the landscape on genetic structure of different species (Goldberg & Waits, 2010; Rioux Paquette, Talbot, Garant, Mainguy, & Pelletier, 2014). Comparison of the effects of the landscape on ecologically interacting species has also received some attention (James, Coltman, Murray, Hamelin, & Sperling, 2011), although comparative landscape genetic analysis of hosts and parasites is so far limited. While it is often assumed that genetic structure in parasites is correlated with dispersal patterns of their hosts, the strength of this correlation varies with several factors such as difference in generation time, degree of generalism of the parasite, and proportion of the life cycle of the parasite spent free from the host (Mazé‐Guilmo, Blanchet, McCoy, & Loot, 2016). Even if a parasite depends entirely on the host for dispersal, specific details of how transmission and movement between host individuals occurs can lead to differences between parasite and host in genetic structure and dispersal patterns. For example, there is a discrepancy between patterns of relatively strong genetic structure in a human roundworm parasite, which transmits through human feces, and extensive movement in their human host. This discrepancy may be explained by the fact that the parasites transmit between host individuals during defecation, which primarily occurs within human households, resulting in parasite gene flow that is spatially restricted (Criscione et al., 2010). If transmission of parasites among host individuals occurs in environments that are not the most conducive to host dispersal and gene flow, then the effects of land cover on genetic structure may differ between the parasite and its hosts. However, potentially contrasting effects of the landscape on genetic structure of parasites and hosts have not been described. Here, we analyze and compare the effect of landscape composition on the genetic structure of an ectoparasite and one of its host species.

**Figure 1:**
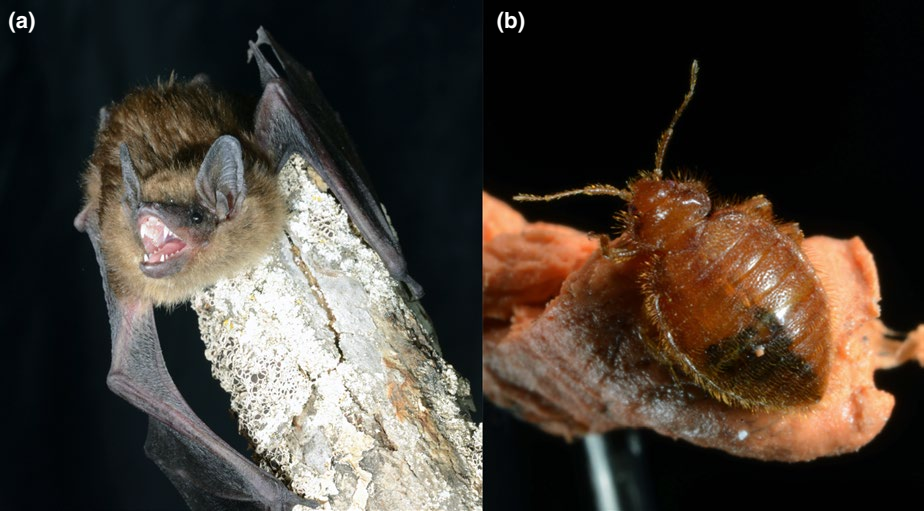
Photograph of a (a) big brown bat and a (b) *Cimex adjunctus* ectoparasite taken by Brock Fenton

Big brown bats (*Eptesicus fuscus*; Figure 1) are native to most of North America, being absent only in northern and eastern regions of Canada. They overwinter in underground openings (caves or mines) or buildings (Whitaker & Gummer, 1992), and roost in attics of buildings (Ellison, O'Shea, Neubaum, & Bowen, 2007) or in trees (Arnett & Hayes, 2009; Willis, Kolar, Karst, Kalcounis‐Rueppell, & Brigham, 2003) in the summer. They forage widely over a range of land cover types with foraging activity occurring mainly in wetlands and developed areas (Furlonger, Dewar, & Fenton, 1987; Lookingbill et al., 2010), although males show lesser foraging site fidelity than females (Wilkinson & Barclay, 1997). While foraging, they often pause in structures, including under bridges, with other individuals and other species before resuming foraging activity (Adam & Hayes, 2000). Generation time in big brown bats is between one and 2 years, depending on location and sex (Kurta & Baker, 1990). In early fall, bats from many summer roosts congregate at the entrance of winter hibernacula and copulate before hibernation, a process known as autumnal swarming (Kurta, 1995). Therefore, gene flow in big brown bats occurs partly in the fall. Gene flow may also occur in the spring, when a small proportion of individuals return to a different summer roost than the one they occupied in the previous year, and during the summer, when some individuals switch summer roosts (Ellison et al., 2007; Willis & Brigham, 2004). Males are thought to disperse among roosts during the summer more frequently than females (Vonhof, Strobeck, & Fenton, 2008). Gene flow in big brown bats may be relatively high, as suggested by low genetic differentiation across North America observed in two studies (Nadin‐Davis, Feng, Mousse, Wandeler, & Aris‐Brosou, 2010; Turmelle, Kunz, & Sorenson, 2011). Nonetheless, gene flow also appears to be limited at larger distances. In a study in eastern Illinois and western Indiana (Vonhof et al., 2008), a significant isolation‐by‐distance (IBD) pattern was observed using microsatellite markers among six big brown bat summer maternity colonies, at an average distance of 54 km from each other. In addition to geographic distance, landscape features such as land cover composition could affect gene flow that results from big brown bat movements among summer roosts and also between summer roosts and hibernacula. Big brown bats are known to avoid field interiors and preferentially move along edges created by either forests or man‐made structures, as do several other bat species including the little brown myotis, the northern myotis, the silver‐haired bat, the hoary bat, the pipistrelle, and the serotine (Jantzen & Fenton, 2013; Verboom & Huitema, 1997). Analysis of the associations between land cover and genetic structure may reveal additional effects of the landscape on gene flow of big brown bats.

Big brown bats are also one of the key hosts of *Cimex adjunctus* (Figure 1), a widespread blood‐feeding insect (Family Cimicidae) that is an ectoparasite of bats in North America. This insect occurs from the eastern seaboard to the Rocky Mountains and from Labrador and the Northwest Territories south to Texas (Usinger, 1966). *Cimex adjunctus* is an ectoparasite of warm‐blooded animals, almost exclusively associated with bats, and is known to be a weak generalist, meaning that it associates with host species that are phylogenetically closely related to each other (Mazé‐Guilmo et al., 2016). *Cimex adjunctus* parasitizes several other bat species in central and eastern North America, and although the full breadth of potential host species is not known, it includes the little brown myotis (*Myotis lucifugus*) and the northern myotis (*Myotis septentrionalis*) (Talbot, Vonhof, Broders, Fenton, & Keyghobadi, 2016; Usinger, 1966). According to Usinger (1966), cimicid ectoparasites associated with bats may display between one and two generations per year, depending on the location. This parasite typically remains in the hosts' roosts, emerging from cracks in the walls to obtain blood meals (Usinger, 1966). It is hypothesized to have limited inherent capacity for movement outside of roosts such that dispersal occurs primarily via individuals being carried by the host (Usinger, 1966). Mist‐net captures of bats transporting *C. adjunctus* (Talbot et al., 2016) confirm this mode of dispersal. Therefore, gene flow in *C. adjunctus* is likely mediated by its bat hosts.

Roost‐switching by bats in the summer is one very possible mechanism by which gene flow in both *C. adjunctus* and the hosts would occur. Whether *C. adjunctus* gene flow can occur during movements between summer roosts and winter hibernacula of bats is less clear because the extent to which *C. adjunctus* overwinters in hibernacula is not known. Gene flow in *C. adjunctus* may also occur during bat foraging; movement of parasites between host individuals could occur at temporary night roosting areas, where bats from different summer day roosts congregate between bouts of feeding (Adam & Hayes, 2000). Therefore, foraging movements of bats, although they do not result in bat gene flow, may affect gene flow in *C. adjunctus*. This is one possible mechanism by which discrepancies in gene flow patterns between bats and *C. adjunctus* could arise. While gene flow in *C. adjunctus* is potentially mediated by multiple bat species, the big brown bat is one of the most common and widespread hosts. Furthermore, key aspects of bat ecology that may contribute to ectoparasite gene flow are shared among several of *C. adjunctus*' hosts. For example, the use of edges at forests and developed areas for movement is common to many bat species (Jantzen & Fenton, 2013; Verboom & Huitema, 1997), as is the use of temporary roosting sites during foraging (Adam & Hayes, 2000). Wetlands are also important sites of foraging activity for several other bat species including the eastern red bat (*Lasiurus borealis*), tri‐colored bat (*Perimyotis subflavus*), and little brown myotis (*M. lucifugus*) (Lookingbill et al., 2010).

In our study, we compared the effects of landscape composition on genetic differentiation in big brown bats and in its parasite *C. adjunctus*. We hypothesized that gene flow of big brown bats preferentially occurs through land cover types that are known to facilitate movement, such as developed or forested areas. We, therefore, predicted a negative effect of these lands covers types on bat genetic differentiation. We also hypothesized that bat gene flow is not associated with open land covers that are either avoided, such as open areas, or used primarily for foraging, such as wetlands, and predicted a neutral or positive effect of these land covers types on bat genetic differentiation. For *C. adjunctus,* we hypothesized that some portion of gene flow occurs during bat foraging, which does not result in gene flow in the bat itself. We therefore predicted that genetic differentiation of the two species could be affected differently by land cover, with a potentially significant negative effect of bat foraging areas, such as wetlands, on genetic differentiation of *C. adjunctus*.

## 2 MATERIALS AND METHODS

### 2.1 Sample collection

**Figure 2:**
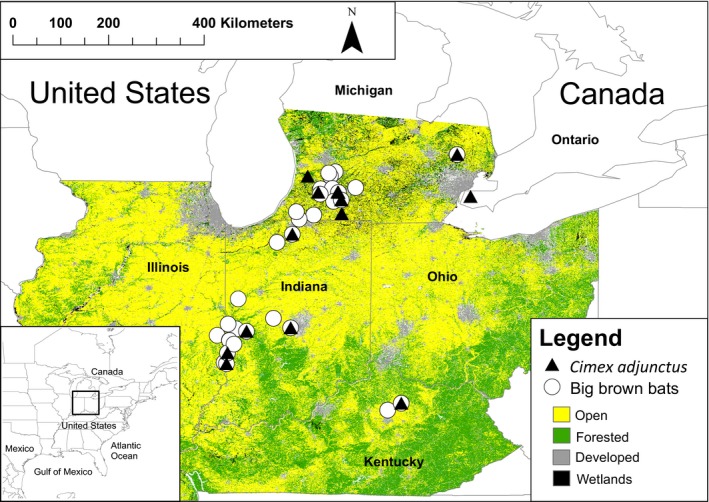
Study area in the southern Great Lakes region of North America. White circles show sampling locations for the big brown bat, *Eptesicus fuscus,* and black triangles show sampling locations for its cimicid ectoparasite, *Cimex adjunctus*. Each of the four land cover types analyzed in our study is shown by a different color

We collected 2‐mm wing biopsies from 142 big brown bats caught in mist‐nets or harp traps in the southern Great Lakes region (Figure 2) between 1997 and 2010. Some of these samples were also used in Vonhof et al. (2008). Upon collection, samples were immediately stored in a 95% ethanol solution until further analysis.

We also collected 55 samples of *C. adjunctus* in the southern Great Lakes region (Figure 2), from 2005 to 2014, that represents a portion of the samples used in Talbot et al. (2016). We removed all but six samples directly from mist‐netted *E. fuscus* host individuals. Mist‐net capture locations were adjacent to a known summer roost (house, barn, church, or school) of *E. fuscus*, or within forested national, provincial, state, or territorial lands (Talbot et al., 2016). Most mist‐netted bats and the *C. adjunctus* individuals they harbored likely came from the adjacent known roost, although it is possible that a small proportion came from different roosts in the area. Overall, between 3% and 15% of mist‐netted bats harbored a parasite, depending on the location. We also sampled six *C. adjunctus* individuals from the interior of a summer roost, in a house attic inhabited by *E. fuscus* (Talbot et al., 2016). Because we could be certain of the roost site in this case, we considered this sampling location as distinct from its adjacent mist‐netting capture location.

### 2.2 Genetic analyses

We genotyped big brown bats at eight microsatellite loci, originally developed for a range of bat species (MMG9 and MM25, from Castella & Ruedi, 2000; TT20 from Vonhof, Davis, Strobeck, & Fenton, 2001; EF1, EF6, EF14, EF15, and EF20 from Vonhof, Davis, Fenton, & Strobeck, 2002). For samples that were also analyzed by Vonhof et al. (2008), we used the genotype data reported in that paper. For all additional samples, we extracted DNA from wing biopsies using the DNeasy Blood and Tissue Kit (QIAGEN, Germantown, MD, USA) and genotyped each sample at the eight microsatellite loci using PCR chemistry and cycling conditions as in Vonhof et al. (2002). We used a DNAEngine Premium Thermal Cycler 200 (BIO‐RAD, Hercules, CA, USA) to execute the polymerase chain reaction (PCR) amplification. We visualized PCR products with 1.5% agarose gel electrophoresis using SYBR Green (BIO‐RAD) on a UV transluminator to check the quality and size of amplified fragments. We then sized products on a 3730xl DNA Analyzer (Applied Biosystems, Foster City, CA, USA).

Samples of *C. adjunctus* were previously genotyped at seven microsatellite loci, originally developed for *C. lectularius* (Cle002, Cle003, Cle013, and Cle015 from Fountain, Duvaux, Horsburgh, Reinhardt, & Butlin, 2014; Clec15, Clec104, and BB28B from Booth et al., 2012), as described in Talbot et al. (2016). We called microsatellite genotypes for each species using ABI's GeneMapper Software v.4.0, and we checked all genotype calls manually.

### 2.3 Statistical analyses

#### 2.3.1 Hardy–Weinberg, linkage disequilibrium and genetic diversity

For each species separately, we used Genepop v4.2 (Raymond & Rousset, 1994) to test for Hardy–Weinberg and linkage disequilibrium in all sites with more than one individual sampled. We corrected *p*‐values for multiple hypothesis testing with Bonferroni correction and used a threshold α of .05. Also, we calculated genetic diversity indices (total number of alleles, average observed and expected heterozygosity, and inbreeding coefficient G_IS_ across sites with more than one individual sampled) for each locus and averaged across all loci.

#### 2.3.2 Comparative effect of geographic distance and land cover

We tested for isolation‐by‐distance (IBD) and effects of landscape composition on genetic differentiation, separately for *C. adjunctus* and the big brown bat, using an individual‐based approach. We used r_W_ (Wang, 2002), calculated with SpaGeDi v1.5 (Hardy & Vekemans, 2002), as a genetic relatedness index. We calculated 1—r_W_ for each pair of individuals of each species to obtain genetic distances. We calculated geographic distance (in km) between sampling locations of individuals, corrected for sphericity of the earth, using the “rdist.earth” function from the “fields” package (Fields Development Team 2006) in R v3.1.3.

Next, to characterize land cover (Table 1) in the southern Great Lakes region of the United States, we used the National Land Cover Database (United States Geological Survey's Land Cover Institute, Sioux Falls, ND, USA). We chose four types of land cover that may affect movements and behaviors of bats: wetland (two types combined: woody and emergent herbaceous), developed, forested (three types combined: deciduous, evergreen, and mixed), and open (four types combined: hay and pasture, cultivated crops, barren land, and grassland). Using ArcGIS v10.3 (ESRI, Redlands, CA, USA), we created a buffer around a straight line between the capture location for each pair of individuals, for both species (Murphy, Dezzani, Pilliod, & Storfer, 2010; Rioux Paquette et al., 2014). We set the buffer's width to 54 km (27 km on either side of the line), the average distance between sampled big brown bat colonies in a previous study in which significant IBD was observed (Vonhof et al., 2008). Using Spatial Analyst (ArcGIS v10.3; ESRI), we calculated the proportion of each land cover type in each linear buffer, corresponding to each pair of individuals.

**Table 1:** Description of each land cover type, from the United States Geological Survey's National Land Cover Database, used in the study, in the southern Great Lakes of North America. The mean proportion (and standard deviation) of each land cover type across all 54‐km wide buffers connecting pairs of samples sites is provided, separately for the big brown bat (*Eptesicus* fuscus) and its cimicid ectoparasite (*Cimex adjunctus*)

Land cover type	Description	Average proportion	
		*Cimex adjunctus*	*Eptesicus fuscus*
Developed	Areas with a mixture of constructed materials and vegetation, where constructed materials account for 30%–100% of the cover, and vegetation accounts for 0%–70% of the cover	0.09 (0.12)	0.04 (0.06)
Forested	Areas dominated by trees generally greater than 5 m tall, and greater than 20% of total vegetation cover	0.12 (0.09)	0.05 (0.09)
Open	Areas of cultivated crops, hay or pasture, dominated by gramanoid or herbaceous vegetation, or barren of any structure or vegetation	0.44 (0.25)	0.68 (0.29)
Wetlands	Areas where the soil or substrate is periodically saturated with or covered with water	0.06 (0.05)	0.02 (0.04)

To compare the effect of landscape composition on genetic differentiation between the parasite and the host, we fit pairwise genetic distance (1 — r_W_), for each species separately, to geographic distance and proportion of each type of land cover using multiple regression on distance matrices with the “MRM” function from the “ecodist” package (Goslee & Urban, 2007) in R v3.1.3. This function determines significance of predictors through permutation (9,999 replicates) of distance matrices (Legendre, Lapointe, & Casgrain, 1994; Lichstein, 2007). We compared models for big brown bats and *C. adjunctus* to determine which land cover types have a significant positive or negative relationship with genetic distance in each of the host and the parasite.

We used an approach based on quantifying land cover composition in broad, linear buffers (Murphy et al., 2010; Rioux Paquette et al., 2014), as opposed to a resistance matrix approach (Spear, Balkenhol, Fortin, McRae, & Scribner, 2010; McRae and Beier 2007), for two reasons. First, our approach is arguably more appropriate for animals that can fly long distances over the landscape or in the case of *C. adjunctus* that are transported by such flying animals. Flying animals such as bats may easily move over smaller areas that are unsuitable or could otherwise represent high resistance (e.g., Amos et al., 2012). As a result, they are likely to respond to the composition of the landscape at a coarser scale rather than to detailed configuration of the landscape, and the paradigm of the resistance surface may not apply as well to such highly mobile, volant animals as it does to less mobile and non‐volant animals. Second, our approach is less dependent on a priori knowledge or hypotheses of which landscape elements affect gene flow (Spear et al., 2010), which is particularly important for *C. adjunctus*, a species for which very little is known regarding basic aspects of ecology and movement.

## 3 RESULTS

We obtained genotypes of 142 big brown bat individuals (49 males and 93 females; 114 adults and 28 juveniles), from 32 roosts in the lower Great Lakes region of North America (Appendices S1 and S2). We also obtained genotypes of 55 *C. adjunctus* from 15 roosts (Appendix S3; microsatellite data available in Talbot et al., 2016). The average distance between roosts for big brown bat samples was 141 km (range of 0.001–502 km). The average distance between roosts for *C. adjunctus* samples was 181 km (range of 0.012–511 km).

### 3.1 Hardy–Weinberg, linkage disequilibrium, and genetic diversity

We found no significant evidence, after Bonferroni correction, of Hardy–Weinberg disequilibrium in big brown bats, nor linkage disequilibrium in either species. We found three significant cases of deviation from Hardy–Weinberg equilibrium in *C. adjunctus* (one population at Clec104 and Cle015 and another population at Clec104). These incidences of deviation from Hardy–Weinberg equilibrium were not systematic across loci, which would have suggested presence of null alleles, or across populations. Therefore, we retained these two markers and two populations for our analyses. Genetic diversity indices were overall higher in big brown bats than in *C. adjunctus* across microsatellite markers (Table 2), and values in *C. adjunctus* were very similar to those found in a study spanning a slightly larger study area in the same region (Talbot, Vonhof, Broders, Fenton, & Keyghobadi, 2017). Total number of alleles averaged at 28.9 in big brown bats and 5.6 in *C. adjunctus*, across microsatellite markers. Mean observed and expected heterozygosities, averaged across sites and across loci, were 0.815 and 0.861, respectively, in big brown bats and 0.256 and 0.434 in *C. adjunctus*. The mean inbreeding coefficient, averaged across sites and across loci, was 0.053 in big brown bats and 0.433 in *C. adjunctus*. Finally, pairwise genetic distances between individuals (1 — r_W_) across the whole dataset were, on average, lower for big brown bats than for *C. adjunctus* [Big brown bat: 1.01 ± 0.11 (*SD*); *C. adjunctus*: 1.28 ± 0.61 (*SD*)].

**Table 2:** Genetic diversity indices (total number of alleles, *N*_A_, observed and expected heterozygosity, *H*_O_ and *H*_E_, and inbreeding coefficient, *G*_IS_) per microsatellite locus and averaged across loci (Average). Values of *H*_O_, *H*_E,_ and *G*_IS_ are averaged across sites with more than one individual sampled, for big brown bats (*Eptesicus* fuscus; 141 individuals from 31 sites) and its cimicid ectoparasite (*Cimex adjunctus*; 50 individuals from 10 sites)

Species	Locus	*N*_A_	*H*_O_	*H*_E_	*G*_IS_
*Eptesicus fuscus*	EF1	23	0.90	0.89	−0.01
	EF6	30	0.93	0.93	<0.01
	EF14	31	0.87	0.89	0.02
	EF15	38	0.73	0.92	0.20
	EF20	29	0.79	0.90	0.12
	MMG9	46	0.87	0.96	0.09
	MMG25	19	0.63	0.66	0.05
	TT20	15	0.81	0.75	−0.07
	Average	28.9	0.815	0.861	0.053
*Cimex adjunctus*	Clec104	4	0.25	0.45	0.45
	Clec15	3	0.11	0.06	−0.81
	BB28B	4	0.53	0.40	−0.32
	Cle002	5	0.11	0.29	0.61
	Cle013	13	0.31	0.68	0.54
	Cle003	6	0.34	0.60	0.43
	Cle015	4	0.06	0.56	0.89
	Average	5.6	0.256	0.434	0.433

### 3.2 Comparative effect of geographic distance and land cover

In big brown bats, geographic distance, proportion of open land cover, and proportion of developed land cover had significant relationships (Table 3) with genetic distance (final model *R* ^2^ = 0.04; Table 3). Genetic distance showed a positive relationship with both geographic distance (*p *<* *.01) and proportion of open land cover (*p *<* *.01), but a negative relationship with developed land cover (*p *=* *.034). These results suggest that geographic distance and open land cover may act to limit gene flow in big brown bats, while developed lands may facilitate gene flow.

**Table 3:** Effects of geographic distance and four different land cover types (Developed areas, Forested areas, Open areas, and Wetlands) on genetic distance (1 — r_W_, where r_w_ is the relatedness coefficient of Wang, 2002) between individuals in the big brown bat (*Eptesicus* fuscus) and its cimicid ectoparasite (*Cimex adjunctus*), in the southern Great Lakes region of North America. Proportion of different land cover types were measured in 54‐km wide buffers between each pair of individuals, for each species separately. Models were fit using multiple regression on distance matrices (MRM). *p*‐values for significant effects are bolded

Species	*Cimex adjunctus*	*Eptesicus fuscus*
Number of microsatellite markers	7	8
Sample size	55	142
Geographic distance		
Slope	0.0005	0.0002
*SE*	0.0005	<0.0001
*p*	.111	**<.001**
Developed		
Slope	0.1970	−0.0738
*SE*	0.4089	0.0453
*p*	.567	**.034**
Forested		
Slope	0.9527	−0.0044
*SE*	0.8036	0.0316
*p*	**.021**	.859
Open		
Slope	0.1808	0.0460
*SE*	0.3177	0.0095
*p*	.404	**<.001**
Wetlands		
Slope	−2.2797	−0.0183
*SE*	1.9225	0.0588
*p*	**.040**	.644
Final model		
*R* ^2^	0.06	0.04

In *C. adjunctus*, proportion of forested land cover and proportion of wetlands both had a marginally significant relationship with genetic distance (final model *R* ^2^ = 0.06; Table 3). The effect of forested land cover on genetic distance was positive (*p *=* *.021), while the effect of wetlands was negative (*p *=* *.04). These results suggest that forests may act to limit gene flow in *C. adjunctus* while wetlands may facilitate gene flow.

## 4 DISCUSSION

### 4.1 Effect of land cover on genetic structure of the big brown bat and its ectoparasite

First, our results support an earlier finding by Vonhof et al. (2008) of a significant positive relationship between geographic distance and genetic distance in big brown bats. Concordant with our predictions, we also found a significant effect of two land cover types on genetic structure in big brown bats. It has been suggested that bats preferentially move close to tall structures, either trees or man‐made structures, to avoid energy expenditures associated with moving against high winds (Jantzen & Fenton, 2013). Therefore, open land cover, which represented a very large proportion of our study area, may be avoided. Consistent with this expectation, our results suggest that open land cover may act to limit gene flow in this species. Additionally, our results suggest that developed land cover may facilitate gene flow and support the hypothesis that big brown bats move preferentially along leeward edges of structural features (Jantzen & Fenton, 2013).

Concordant with our predictions, we also found a significant effect of two land cover types, forested and wetlands, on genetic distance in *C. adjunctus*. These were different than the types of land cover found to affect big brown bat genetic distance, even though *C. adjunctus* almost entirely depends on its hosts to move outside of roosts (Usinger, 1966). Furthermore, in contrast to our results on the big brown bat, we did not find IBD in *C. adjunctus*. Overall, our results suggest that a parasite and a host, while linked in their movements, may show differences in gene flow patterns. These differences may at least be partially explained by differences between the two species in the environments and types of land cover in which gene flow occurs. Lookingbill et al. (2010) found the activity of several bat species, including the big brown bat, to be correlated with wetland cover. Our result of a negative effect of wetland cover on *C. adjunctus* genetic distance supports the hypothesis that gene flow in the ectoparasite may occur during foraging by bats in wetlands, possibly via transfer between individuals in temporary, communal roosts.

Our results suggest that forested areas impede gene flow in the ectoparasite *C. adjunctus*. While several bat species are known to move along forest edges, they also show reduced activity in forest interiors and densely vegetated areas (Jantzen & Fenton, 2013; Loeb & O'Keefe, 2006). This restricting effect of contiguous or dense forest cover on bats could explain the positive effect of forest cover on *C. adjunctus* genetic distance. In addition, even when bats do forage in forested areas, it is possible that these environments provide few opportunities for *C. adjunctus* gene flow via transfer between host individuals, if there are few temporary, communal roosting sites for bats. While foraging in these environments, bats may be more likely to temporarily roost by themselves in trees. Finally, it is also possible that *C. adjunctus* experiences higher mortality or removal when bats travel through forested areas, although the exact mechanism by which this might occur is not clear.

Sample sizes in our study are larger for the big brown bat than its parasite. This is a function of the parasite being present on only a subset of sampled host individuals. While our sample sizes for *C. adjunctus* are relatively small, we used an individual‐based analysis, which has been shown to allow for robust landscape genetic inference given small sample sizes (Prunier et al., 2013). Several other studies have used an individual‐based approach with sample sizes similar to ours in drawing population genetic and landscape genetic inferences (Broquet et al., 2006; Laurence, Smith, & Schulte‐Hostedde, 2013).

Finally, more information is needed on the effects of land cover on gene flow in males versus females, and in different age groups, in big brown bats. Sex‐biased dispersal and sex‐biased and age‐biased parasitism, both suggested for big brown bats (Pearce & O'Shea, 2007; Vonhof et al., 2008), are important factors to take into account when comparing gene flow patterns between a host and a parasite.

### 4.2 Correlation between genetic differentiation of a host and a parasite

Although there are many examples in which host and parasite movement or gene flow are correlated (Bruyndonckx, Henry, Christe, & Kerth, 2009; Levin & Parker, 2013; Nieberding, Morand, Libois, & Michaux, 2004; Nieberding et al., 2008), parasites often show higher levels of genetic differentiation than their hosts, possibly because of lower effective population size and shorter generation time in the parasite than the host (Mazé‐Guilmo et al., 2016). For example, higher genetic structure in the trematode parasite (*Pagioporus shawi*) compared to its host, the steelhead trout (*Oncorhynchus mykiss*), led to parasite genotypes providing more accurate population assignments in the host than could be obtained by examining genotypes of the host itself (Criscione, Cooper, & Blouin, 2006). Higher genetic differentiation in a host is also possible. For example, genetic structure among colonies was weaker for fleas than for their prairie dog hosts (Jones & Britten, 2010). In addition to effective population size and generation time, additional factors that may uncouple the genetic structure of parasites from that of their hosts include host mobility, the degree of generalism of the parasite, and the proportion of time spent in free‐living stages by the parasite (Mazé‐Guilmo et al., 2016).

Our results support the pattern of higher differentiation in the parasite, with higher pairwise genetic distances in *C. adjunctus* than in the big brown bat. Two other studies on *C. adjunctus* conducted at two different spatial scales also found a much higher degree of genetic differentiation in the parasite (Talbot et al., 2016, 2017) than has previously been reported in two of its main hosts, the big brown bat (Nadin‐Davis et al., 2010; Vonhof et al., 2008) and little brown myotis (Johnson et al., 2015). This difference was attributed to the fact that *C. adjunctus* is a weak generalist ectoparasite of highly mobile hosts, with a generation time that is likely much shorter than that of its hosts. Results from our landscape analyses suggest that there may be additional differences between *C. adjunctus* and its bat hosts in the location and timing of gene flow that contribute to their different genetic structure.

Although all parasite samples used in this study came from the body of big brown bats or in a roost inhabited by big brown bats, *C. adjunctus* can use several different bat species as hosts. In a range‐wide study of the genetic structure of *C. adjunctus*, Talbot et al. (2016) noted moderate differentiation among parasite samples from different host species at microsatellite markers and very little differentiation at mitochondrial DNA. Therefore, individuals of *C. adjunctus* may switch host species somewhat regularly, a situation expected for a generalist ectoparasite. It is possible that the different responses of big brown bats and *C. adjunctus* to landscape composition partly reflect the fact that other bat species, such as *M. lucifugus* or *M. septentrionalis*, are also contributing to *C. adjunctus* gene flow. However, several key aspects of the ecology of big brown bats, including the use of wetlands for foraging, the use of temporary roosts while foraging, and seasonal patterns of gene flow, are shared with other bat species that are potential hosts of *C. adjunctus* (Adam & Hayes, 2000; Lookingbill et al., 2010). As a result, our predictions regarding effects of land cover on *C. adjunctus* genetic differentiation arise not just from the behavior of big brown bats, but from the behavior of multiple potential host species. Furthermore, because big brown bats are among the more widely dispersing of *C. adjunctus*' potential hosts, this bat species is likely to determine the upper limit of gene flow, and hence patterns of genetic differentiation, in the parasite.

While the effects of the landscape on gene flow and genetic structure of many animal species have been described (Manel & Holderegger, 2013; Storfer et al., 2010), not much is known about how species that are dependent on the movements of other species, as is the case with many parasites, interact with the landscape (Sprehn, Blum, Quinn, & Heins, 2015). Our study has revealed a difference in the types of land cover that correlate with genetic differentiation of a generalist ectoparasite versus one of its potential bat host species. Our results suggest that in addition to factors such as host mobility, the proportion of time spent in free‐living stages by the parasite, and the generalist nature of the parasite (Mazé‐Guilmo et al., 2016), differences between hosts and parasites in the nature, timing and location of gene flow events can also lead to discordant patterns of genetic structure.

## CONFLICT OF INTEREST

None declared.

## AUTHOR'S CONTRIBUTIONS

BT's work involved conception of the study, collection of some samples, execution of genetic and statistical analyses, and writing the first draft. NK coordinated the study, supervised the collection and interpretation of genetic data, and revised the writing. BF contributed to the collection of data, coordinated the study, supervised the interpretation of data, and revised the writing. MJV and HGB contributed with most of the sample collection and helped in the interpretation of data and revision of the writing.
